# Optimization of the MALDIxin test for the rapid identification of colistin resistance in *Klebsiella pneumoniae* using MALDI-TOF MS

**DOI:** 10.1093/jac/dkz405

**Published:** 2019-10-03

**Authors:** Laurent Dortet, Agnieszka Broda, Sandrine Bernabeu, Youri Glupczynski, Pierre Bogaerts, Rémy Bonnin, Thierry Naas, Alain Filloux, Gerald Larrouy-Maumus

**Affiliations:** 1 MRC Centre for Molecular Bacteriology and Infection, Department of Life Sciences, Faculty of Natural Sciences, Imperial College London, London, SW7 2AZ, UK; 2 Department of Bacteriology-Hygiene, Bicêtre Hospital, Assistance Publique - Hôpitaux de Paris, Le Kremlin-Bicêtre, France; 3 EA7361 ‘Structure, Dynamic, Function and Expression of Broad Spectrum β-Lactamases’, Paris-Sud University, LabEx Lermit, Faculty of Medicine, Le Kremlin-Bicêtre, France; 4 French National Reference Centre for Antibiotic Resistance, Le Kremlin-Bicêtre, France; 5 Laboratory of Clinical Microbiology, Belgian National Reference Centre for Monitoring Antimicrobial Resistance in Gram-Negative Bacteria, CHU UCL Namur, Yvoir, Belgium

## Abstract

**Background:**

With the dissemination of carbapenemase producers, a revival of colistin was observed for the treatment of infections caused by MDR Gram-negatives. Unfortunately, the increasing usage of colistin led to the emergence of resistance. In *Klebsiella pneumoniae*, colistin resistance arises through addition of 4-amino-l-arabinose (l-Ara4N) or phosphoethanolamine (pEtN) to the native lipid A. The underlying mechanisms involve numerous chromosome-encoded genes or the plasmid-encoded pEtN transferase MCR. Currently, detection of colistin resistance is time-consuming since it still relies on MIC determination by broth microdilution. Recently, a rapid diagnostic test based on MALDI-TOF MS detection of modified lipid A was developed (the MALDIxin test) and tested on *Escherichia coli* and *Acinetobacter baumannii*.

**Objectives:**

Optimize the MALDIxin test for the rapid detection of colistin resistance in *K. pneumoniae*.

**Methods:**

This optimization consists of an additional mild-acid hydrolysis of 15 min in 1% acetic acid. The optimized method was tested on a collection of 81 clinical *K. pneumoniae* isolates, including 49 colistin-resistant isolates (45 with chromosome-encoded resistance, 3 with MCR-related resistance and 1 with both mechanisms).

**Results:**

The optimized method allowed the rapid (<30 min) identification of l-Ara4N- and pEtN-modified lipid A of *K. pneumoniae*, which are known to be the real triggers of polymyxin resistance. At the same time, it discriminates between chromosome-encoded and MCR-related polymyxin resistance.

**Conclusions:**

The MALDIxin test has the potential to become an accurate tool for the rapid determination of colistin resistance in clinically relevant Gram-negative bacteria.

## Introduction

Currently, antimicrobial resistance is at the top of the agenda for scientists and governments, while XDR organisms, such as carbapenemase-producing Enterobacterales (CPE) are rapidly emerging. The pipeline of new antibiotics is very limited, and colistin is now considered as one of the last-resort therapies for the treatment of infection caused by XDR Gram-negative bacteria.^1^ In countries that are considered to be endemic for CPE (e.g. Greece, Italy), colistin is often used as empirical treatment for severe infection, such as bacteraemia. Unfortunately, this increased use of colistin in the therapeutic armamentarium has led inexorably to the development of resistance.^2–6^

In Gram-negative bacteria, acquired resistance to colistin results mostly from modifications of the drug target, i.e. LPS. These modifications correspond to addition(s) of 4-amino-l-arabinose (l-Ara4N) and/or phosphoethanolamine (pEtN) to lipid A, the anchor of LPS. Addition of such cationic components leads to the repulsion of colistin (an old class of cationic antibiotic that targets polyanionic bacterial LPS and disrupts the bacterial outer membranes), resulting in the protection against outer-membrane disruption by the antibiotic.^7^^,^^8^ The mechanisms involved in such modification of lipid A might be chromosome or plasmid encoded. Plasmid-encoded resistance to colistin involves the acquisition of an *mcr*-like gene encoding a specific pEtN transferase.^9^ MCR producers have mostly been reported among *Escherichia coli* and *Salmonella* spp.^9^ In contrast, in *Klebsiella* spp., chromosome-encoded resistance has been reported to be far more prevalent than *mcr* acquisition. The most prevalent chromosome-encoded mechanisms are mutations in genes encoding the PmrA/PmrB or PhoP/PhoQ two-component systems and (even more prevalent) alterations of the master regulator MgrB.^10^

Although the epidemiology of acquired colistin resistance varies depending on the bacterial species and geographical area, rapid detection of such resistance is one of the key ways of improving the treatment of patients infected with MDR bacteria for which other alternatives are not available (e.g. MBL producers). Currently, detection of colistin resistance in Enterobacterales relies on MIC determination using broth microdilution, which is the gold standard for colistin susceptibility testing.^11^ Recently, we developed a novel rapid approach using MALDI-TOF MS that detects colistin resistance directly on intact bacteria in <15 min, the MALDIxin test.^12^ It has been validated for *E. coli* and *Acinetobacter baumannii*, for which it can discriminate between chromosome- and/or plasmid-encoded resistance (i.e. *mcr*), besides detecting colistin resistance.^12^^,^^13^

Here we report an optimization of the MALDIxin test for the rapid detection of colistin resistance in *Klebsiella pneumoniae*.

## Materials and methods

### Bacterial isolates

A collection of 81 *K. pneumoniae* clinical isolates from Belgian and French national reference centres for antimicrobial resistance were used in this study (Table 1), including 49 colistin-resistant isolates (45 with chromosome-encoded resistance, 3 with MCR-related resistance and 1 with both mechanisms) and 32 colistin-susceptible isolates.

**Table 1.** Results of the MALDIxin test on *K. pneumoniae* clinical isolates

Isolate name	Colistin MIC (mg/L)	Resistance mechanism to polymyxins	β-Lactam resistance mechanisms (ESBLs, carbapenemases, …)	Percentage modified lipid A^a^	Percentage pEtN^a^	Percentage l-Ara4N^a^	Reference
Colistin-susceptible isolates							
1609079984	0.5		WT	0.0±0.0	0.0±0.0	0.0±0.0	this study
1609056413	0.5		WT	0.0±0.0	0.0±0.0	0.0±0.0	^17^
1609078951	0.5		WT	0.0±0.0	0.0±0.0	0.0±0.0	this study
1609078870	0.5		WT	0.0±0.0	0.0±0.0	0.0±0.0	this study
1609068884	0.5		WT	0.0±0.0	0.0±0.0	0.0±0.0	this study
1609059262	0.5		WT	0.0±0.0	0.0±0.0	0.0±0.0	this study
1609061149	1		WT	0.0±0.0	0.0±0.0	0.0±0.0	^17^
1609072327	0.5		WT	0.0±0.0	0.0±0.0	0.0±0.0	this study
1609075598	0.5		WT	0.0±0.0	0.0±0.0	0.0±0.0	this study
1609071256	0.5		WT	0.0±0.0	0.0±0.0	0.0±0.0	this study
2 E8	0.5		CTX-M-3	0.0±0.0	0.0±0.0	0.0±0.0	^17^
2 F1	0.5		CTX-M-14	0.0±0.0	0.0±0.0	0.0±0.0	^17^
2 F4	0.5		CTX-M-15	0.0±0.0	0.0±0.0	0.0±0.0	^18^
2 F5	0.5		CTX-M-15	0.0±0.0	0.0±0.0	0.0±0.0	^18^
2 I5	0.5		CTX-M-15 + TEM-1 + SHV-11	0.0±0.0	0.0±0.0	0.0±0.0	^17^
3 C4	0.5		CTX-M-15 + TEM-1B + SHV-11	0.0±0.0	0.0±0.0	0.0±0.0	this study
3 D6	0.5		CTX-M-15 + TEM-1B + SHV-28	0.0±0.0	0.0±0.0	0.0±0.0	this study
3 D7	0.5		CTX-M-15 + TEM-1B + SHV-83	0.0±0.0	0.0±0.0	0.0±0.0	this study
2 I3	0.5		CTX-M-15 + TEM-1 + SHV-11	0.0±0.0	0.0±0.0	0.0±0.0	^18^
2 I4	0.5		CTX-M-15 + TEM-1 + SHV-11	0.0±0.0	0.0±0.0	0.0±0.0	^17^
1 F7	0.5		**KPC-2** + SHV-11 + CTX-M-15	0.0±0.0	0.0±0.0	0.0±0.0	this study
3 B4	0.5		**KPC-3** + TEM-1A, OXA-9, SHV-11	0.0±0.0	0.0±0.0	0.0±0.0	^17^
3 B7	0.5		**GES-5** + SHV-12	0.0±0.0	0.0±0.0	0.0±0.0	^17^
1 B6	0.5		**NDM-1** + TEM-1 + CTX-M-15 + SHV-12 + OXA-9	0.0±0.0	0.0±0.0	0.0±0.0	^17^
1 C9	0.5		**VIM-**1 + SHV-5	0.0±0.0	0.0±0.0	0.0±0.0	^17^
1 E3	0.5		**IMP-1** + TEM-15 + CTX-M-15	0.0±0.0	0.0±0.0	0.0±0.0	this study
2 B1	0.5		**OXA-48**	0.0±0.0	0.0±0.0	0.0±0.0	^18^
2 C6	0.5		**NDM-1** + **OXA-181** + CTX-M-15 + TEM-1 + OXA-1	0.0±0.0	0.0±0.0	0.0±0.0	^17^
2 D2	0.5		**OXA-204** + CMY-4	0.0±0.0	0.0±0.0	0.0±0.0	^17^
2 D8	0.5		**OXA-232** + TEM-1 + CTX-M-15 + OXA-1	0.0±0.0	0.0±0.0	0.0±0.0	^18^
S15	0.25	*mgrB* truncated in ORF by IS*Kpn25* + PhoP (I201N)	**KPC-2** + OXA-9 + TEM-1	0.0±0.0	0.0±0.0	0.0±0.0	^19^
S17	0.5	*mgrB* truncated in ORF by IS*Kpn25* + ArnC (C161Y)	**KPC-2** + OXA-9 + TEM-1	0.0±0.0	0.0±0.0	0.0±0.0	^19^
Chromosome-encoded resistance							
TUN-ST-15	>128	frameshift *mgrB*	CTX-M-15 + OXA-1 + SHV-28	10.6±1.5	0.0±0.0	100±0.0	^20^
TUN-ST-101	8	frameshift *mgrB*	**OXA-48** + CTX-M-15 + OXA-1	19.5±6.6	0.0±0.0	100±0.0	^20^
CNR 111 C2	16	frameshift *mgrB*	**OXA-48** + BLSE	17.0±5.0	0.0±0.0	100±0.0	this study
CNR 20140042	16	MgrB N42Y and K43I	**OXA-48 type** + CTX-M grp1	18.9±3.4	0.0±0.0	100±0.0	^17^
CNR 20140423	32	MgrB N42Y and K43I	**OXA-48 type** + CTX-M grp1	26.5±7.6	0.0±0.0	100±0.0	this study
CNR 20140661	64	MgrB Q30 stop	**KPC type**	29.7±4.6	0.0±0.0	100±0.0	^17^
CNR 20150586	64	MgrB Q30 stop	ESBL	15.0±3.7	0.0±0.0	100±0.0	this study
CNR 20150324	32	MgrB Q30 stop	ESBL	11.8±1.8	0.0±0.0	100±0.0	this study
CNR 20150655	64	MgrB Q30 stop	CTX-M grp1	15.8±7.5	0.0±0.0	100±0.0	this study
CNR 20150542	32	MgrB L4 stop	**OXA-48 type** + CTX-M grp1	15.2±5.7	0.0±0.0	100±0.0	this study
CNR 20151119	64	MgrB L4 stop	**OXA-427**	16.7±3.8	0.0±0.0	100±0.0	^17^
CNR 20150622	64	MgrB Y41 stop	**KPC type** + ESBL	27.6±11.4	0.0±0.0	100±0.0	^17^
CNR 20150777	128	MgrB Y41 stop	CTX-M grp1	27.2±9.2	0.0±0.0	100±0.0	^17^
CNR 20140698	32	MgrB modified from AA 42 (I)	**KPC type**	31.1±9.9	0.0±0.0	100±0.0	this study
CNR 20150944	64	MgrB modified from AA 42 (I)	**KPC type**	42.6±8.4	0.0±0.0	100±0.0	^17^
CNR 20150309	64	MgrB modified from AA 37 (V)	**KPC type** + CTX-M grp1	18.7±3.9	0.0±0.0	100±0.0	^17^
CNR 20150675	64	*mgrB* truncated in ORF by IS*10*	**OXA-427** + CTX-M grp1	30.6±8.6	0.0±0.0	100±0.0	^17^
CNR 20150276	32	*mgrB* truncated in ORF by IS*10*	**OXA-427** + CTX-M grp1	34.8±12.0	0.0±0.0	100±0.0	this study
CNR 20140483	32	*mgrB* truncated in ORF by IS*1R*-like	**OXA-48 type** + CTX-M grp1	28.3±7.5	0.0±0.0	100±0.0	^17^
CNR 20140563	64	*mgrB* truncated in ORF by IS*1R*	**OXA-48 type** + DHA type	31.7±9.3	0.0±0.0	100±0.0	^17^
CNR 20150295	8	*mgrB* truncated in ORF by IS*1R*	**OXA-48 type** + CTX-M grp1	16.7±4.5	0.0±0.0	100±0.0	this study
CNR 20150713	64	*mgrB* truncated in ORF by IS*1R*	**OXA-48 type** + CTX-M grp1	20.9±3.4	0.0±0.0	100±0.0	this study
S20-003	64	*mgrB* truncated in ORF by IS*1R*	**OXA-48 type**	17.3±4.3	0.0±0.0	100±0.0	this study
CNR 20150736	32	*mgrB* truncated in ORF by IS*1R*	**OXA-48 type** + CTX-M grp1	34.0±4.3	0.0±0.0	100±0.0	this study
CNR 20150960	32	*mgrB* truncated in ORF by IS*1R*	ND	16.8±2.9	0.0±0.0	100±0.0	this study
CNR 20151181	32	*mgrB* truncated in ORF by IS*1R*	**OXA-48 type** + CTX-M grp1	247.6±8.9	0.0±0.0	100±0.0	this study
CNR 20150050	32	*mgrB* truncated in promoter by IS*1R*	DHA type	22.1±5.9	0.0±0.0	100±0.0	^17^
CNR 20150943	32	*mgrB* truncated in promoter by IS*1R*	DHA type	26.8±11.7	0.0±0.0	100±0.0	^17^
CNR 20140591	64	*mgrB* truncated in ORF by IS*5*-like	**KPC type** + ESBL	31.3±11.0	0.0±0.0	100±0.0	^17^
CNR 20140862	16	*mgrB* truncated in ORF by IS*5*-like	ND	23.9±6.5	0.0±0.0	100±0.0	this study
CNR 20150573	32	*mgrB* truncated in ORF by IS*5*-like	**NDM type** + **OXA-48 type** + CTX-M grp1	38.4±25.4	0.0±0.0	100±0.0	this study
CNR 20140550	32	*mgrB* truncated in promoter by IS*903D*	**KPC type** + CTX-M grp9	31.9±8.6	0.0±0.0	100±0.0	this study
CNR 20151285	32	*mgrB* truncated in ORF by IS*903*-like	CTX-M grp1	21.3±10.9	0.0±0.0	100±0.0	^17^
S12-172	32	*mgrB* truncated in ORF by IS*903*-like	**NDM type**	29.6±4.1	0.0±0.0	100±0.0	this study
S14-002	64	*mgrB* truncated in promoter by IS*Kpn14*	**KPC type**	44.2±19.2	0.0±0.0	100±0.0	^17^
CNR 20140101	32	*ΔmgrB*	**OXA-48 type** + CTX-M grp1	23.4±5.3	0.0±0.0	100±0.0	^17^
CNR 20150078	32	*ΔmgrB*	**OXA-48 type** + CTX-M grp9	20.1±4.7	0.0±0.0	100±0.0	^17^
CNR 20150066	16	*ΔmgrB*	**OXA-48 type** + BLSE	23.4±3.8	0.0±0.0	100±0.0	^17^
CNR 20151223	32	*ΔmgrB*	carbapenem resistant via impermeability	51.3±8.6	0.0±0.0	100±0.0	^17^
S1	128	*mgrB* truncated in ORF by IS*Kpn25*	**KPC-2** + OXA-9 + TEM-1	40.9±13.6	0.0±0.0	100±0.0	^19^
S12	64	*mgrB* truncated in ORF by IS*Kpn25*	**KPC-2** + OXA-9 + TEM-1	26.6±3.8	0.0±0.0	100±0.0	^19^
CNR 1630	32	*mgrB* truncated in ORF by IS5	ND	27.2±9.1	0.0±0.0	100±0.0	^17^
CNR 1631	8	*mgrB* truncated in ORF by IS5	ND	42.5±24.7	0.0±0.0	100±0.0	this study
CNR 1795	128	*mgrB* truncated in ORF by IS5	ND	34.0±4.3	0.0±0.0	100±0.0	this study
CNR 1861	16	mutated PmrB (T157P)	ND	28.8±5.1	0.0±0.0	100±0.0	^17^
MCR-related resistance to colistin							
CNR 1732	4	*mcr-1*	ND	32.1±7.7	100±0.0	0.0±0.0	^17^
CNR 1853	4	*mcr-1*	ND	34.4±11.8	95.3±6.2	4.7±6.2	^17^
CNR 186 G1	8	*mcr-8*	SHV-27 + TEM-1B + SCO-1	24.4±5.6	100±0.0	0.0±0.0	this study
MCR + chromosome-encoded resistance to colistin							
CNR 1601	32	*mcr-1 + mgrB* truncated in ORF by IS*5*	ND	33.6±19.6	46.1±13.1	53.9±13.1	^17^

ND, not determined.

Carbapenemases are shown in bold and ESBLs are underlined.

a Mean±standard error of the mean.

### Susceptibility testing

Colistin MIC was determined by broth microdilution according to CLSI and EUCAST guidelines.^11^ Results were interpreted using EUCAST breakpoints as updated in 2019 (http://www.eucast.org/clinical_breakpoints/).

### Optimized MALDIxin test

The MALDIxin procedure was performed as previously described,^12^ with the addition of a short mild-acid hydrolysis step, which was crucial for *K. pneumoniae* (Figure S1, available as Supplementary data at *JAC* Online). Briefly, a single colony cultured on Mueller–Hinton agar (bioMérieux, La Balme-les-Grottes, France) was resuspended in 200 μL of distilled water, washed three times with double-distilled water and resuspended in 100 μL of double-distilled water. A 50 μL aliquot was then submitted to mild-acid hydrolysis by the addition of 50 μL of 2% acetic acid in double-distilled water and heating for 15 min at 100°C. The hydrolysed cells were spun, the supernatant was discarded and the pellet was suspended in 25 μL of double-distilled water. An aliquot of 0.4 μL of the bacterial solution was loaded onto the target and immediately overlaid with 0.8 μL of a super-2,5-dihydroxybenzoic acid matrix (Sigma–Aldrich, Gillingham, UK) used at a final concentration of 10 mg/mL in chloroform/methanol (90:10, v/v). Bacterial solution and matrix were mixed directly on the target by pipetting and the mix was dried gently under a stream of air (<1 min). MALDI-TOF MS analysis was performed on a 4800 Proteomics Analyzer (Applied Biosystems, Foster City, CA, USA) using the reflectron mode. Samples were analysed by operating at 20 kV in the negative ion mode using an extraction delay time set at 20 ns. MS data were analysed using Data Explorer version 4.9 (Applied Biosystems).

### Statistical analysis

All experiments were carried out on three independent bacterial cultures. Data were compared two-by-two using the unpaired Welch’s *t-*test. *P* values <0.05 were considered statistically different.

## Results

### Detection of colistin resistance markers in K. pneumoniae using the MALDIxin test

**Figure 1.**
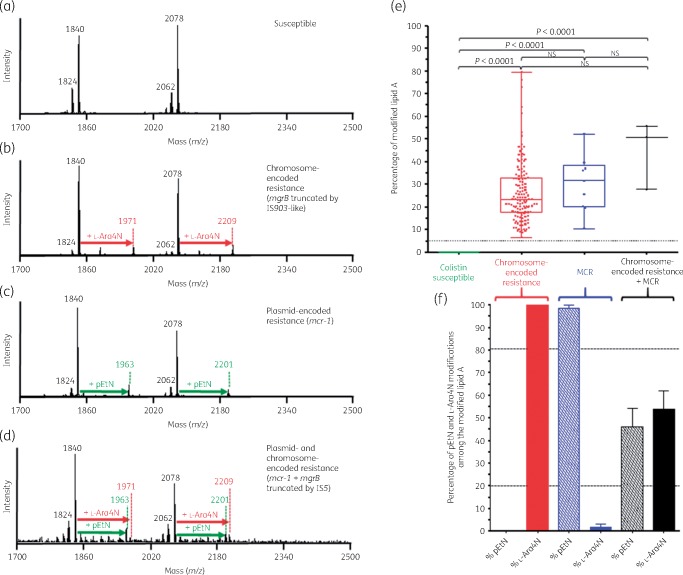
Results of the optimized MALDIxin test on *K. pneumoniae*. Representative spectra of: (a) a polymyxin-susceptible *K. pneumoniae* isolate; (b) a chromosome-encoded (*mgrB* disruption) polymyxin-resistant *K. pneumoniae* isolate; (c) a polymyxin-resistant *K. pneumoniae* isolate producing MCR-1; and (d) a polymyxin-resistant *K. pneumoniae* harbouring both chromosome-encoded resistance (*mgrB* disruption) and plasmid-encoded MCR-1. The peaks at *m/z* 1824, 1840, 2062 and 2078 (black) correspond to the peaks of native *K. pneumoniae* lipid A, the peaks at *m/z* 1971 and 2209 (red) correspond to the addition of l-Ara4N to the native lipid A and the peaks at *m/z* 1963 and 2201 (green) correspond to the addition of one pEtN to the native lipid A. (e and f) Representation of the percentage of the modified lipid A for colistin-susceptible and colistin-resistant *K. pneumoniae* isolates. (e) The global percentage of modified lipid A (l-Ara4N+pEtN-modified lipid A/native lipid A) is represented for colistin-susceptible isolates (*n*=32), chromosome-encoded colistin-resistant *K. pneumoniae* isolates (*n*=45), MCR-producing colistin-resistant *K. pneumoniae* isolates (*n*=3) and the *K. pneumoniae* isolate harbouring both mechanisms (*n*=1). All experiments were performed in triplicate. The error bars represent the standard error of the mean and the dotted horizontal line corresponds to the suggested cut-off for colistin susceptibility related to no modification of lipid A. NS, not significant. (f) Representation of the percentage of l-Ara4N- and pEtN-modified lipid A among the global modified lipid A for colistin-resistant *K. pneumoniae* isolates. The dotted horizontal lines correspond to the proposed cut-offs for discriminating between chromosome-encoded resistance, MCR production and both mechanisms.

In polymyxin-susceptible *K. pneumoniae* isolates, the mass spectrum is dominated by two sets of peaks centred at *m/z* 1840 and *m/z* 2078 (Figure 1a). The ions at *m/z* 1824 and *m/z* 1840 are assigned to a bis-phosphorylated, hexa-acylated lipid A molecule containing or not containing a hydroxylation on the C′-2 fatty acyl chain. The ions at *m/z* 2062 and *m/z* 2078 are assigned to a bis-phosphorylated, hepta-acylated lipid A molecule either containing or not containing a hydroxyl group, respectively, on the C′-2 fatty acyl chain and resulting from a palmitoylation at the C-1 acyl-oxo-acyl position of the molecule at *m/z* 1824 and *m/z* 1840, respectively.

In chromosome-encoded colistin-resistant *K. pneumoniae* isolates, the mass spectrum exhibits two sets of peaks centred at *m/z* 1971 and *m/z* 2209, corresponding to the previously observed *m/z* +131 shifts of mass unit caused by the addition of l-Ara4N to the hexa-and hepta-acylated lipid A structures at *m/z* 1840 and *m/z* 2078, respectively (Figure 1b).

In MCR producers, the mass spectrum exhibits two sets of peaks centred at *m/z* 1963 and *m/z* 2201, corresponding to the previously observed *m/z* +123 shifts of mass unit caused by the addition of pEtN to the hexa-and hepta-acylated lipid A structures at *m/z* 1840 and *m/z* 2078, respectively (Figure 1c).

In colistin-resistant isolates that exhibit both plasmid (*mcr*)- and chromosome-encoded resistance, the mass spectrum exhibits three sets of peaks centred at *m/z* 1963, *m/z* 2201 and *m/z* 2209, corresponding to the previously observed *m/z* +123 shifts of mass unit caused by the addition of pEtN to the hexa-and hepta-acylated lipid A structures at *m/z* 1840 and *m/z* 2078, respectively, and *m/z* +131 shifts of mass unit caused by the addition of l-Ara4N to the hepta-acylated lipid A structures at *m/z* 2078 (Figure 1d).

To further support this observation, we validated the MALDIxin test on 81 *K. pneumoniae* clinical isolates, including 45 colistin-resistant and 36 colistin-susceptible isolates. The percentage of modified lipid A corresponding to the sum of the intensities of the peaks associated with pEtN modification (*m/z* 1963 and *m/z* 2201) and l-Ara4N modification (*m/z* 1971 and *m/z* 2209) divided by the intensities of the peaks assigned to the native lipid A (*m/z* 1824, *m/z* 1840 and *m/z* 2062) allows accurate distinction between colistin-susceptible and colistin-resistant isolates (Figure 1e). Of note, the peak at *m/z* 2078 was not taken into account in the calculation of the percentage of modified lipid A since this native peak observed in colistin-susceptible isolate spectra might potentially correspond to a peak of modified lipid A resulting from the addition of pEtN plus l-Ara4N to the native bis-phosphorylated, hexa-acylated lipid A (*m/z* 2078=*m/z* 1824 + *m/z* 123 + *m/z* 131). The percentage of modified lipid A was found to be 0 for all colistin-susceptible *K. pneumoniae* isolates, while it was >5 for all colistin-resistant isolates (Table 1 and Figure 1e).

### Discrimination between chromosome-encoded resistance, MCR-related resistance and both mechanisms

In *K. pneumoniae* chromosome-encoded colistin resistance results mostly from addition of l-Ara4N to the native lipid A. In contrast, MCR is known to be a pEtN transferase resulting exclusively in addition of pEtN to the native lipid A. Thus, to distinguish between chromosome-encoded resistance and MCR production, the percentages of pEtN and l-Ara4N modifications (% pEtN and % l-Ara4N) were calculated. The % pEtN and % l-Ara4N correspond to the sum of the intensities of the peaks associated with pEtN modifications (*m/z* 1963 and *m/z* 2201) or l-Ara-4N modifications (*m/z* 1971 and *m/z* 2209) divided by intensities of the peaks associated with both modifications (*m/z* 1963 and *m/z* 2201 for pEtN modifications plus *m/z* 1971 and *m/z* 2209 for l-Ara4N modifications). As shown in Figure 1(f), for all colistin-resistant *K. pneumoniae* isolates with chromosome-encoded mechanisms, lipid A was exclusively modified by addition of l-Ara4N. In contrast, MCR-producing isolates had an average % l-Ara4N of 1.6 (0–14.0) and an average % pEtN of 98.4 (86.0–100). When both colistin resistance mechanisms were expressed (only one isolate), % l-Ara4N was 53.9 (36.4–69.5) and % pEtN was 46.1 (30.5–63.6) (Figure 1f). Accordingly, using our isolate collection, arbitrary cut-off values at 20% and 80% for % l-Ara4N and % pEtN, respectively, might be suggested to easily discriminate chromosome-encoded resistance from MCR production and co-expression of both mechanisms.

## Discussion

Here we optimized the MALDIxin test for the detection of colistin resistance in *K. pneumoniae*. The procedure used included a preliminary short (15 min) mild-acid hydrolysis step, which allowed the rapid identification of l-Ara4N- and pEtN-modified lipid A, which are known to be the real triggers of colistin resistance. In *K. pneumoniae*, chromosome-encoded resistance is more frequent than MCR plasmid-encoded resistance. It mainly involves alteration of MgrB, leading to activation of the *arn* operon and subsequent addition of l-Ara4N to the native lipid A.^10^ This modification results in an *m/z* +131 shift of the native lipid A-related peaks. In contrast, expression of MCR enzymes results in the addition of pEtN to the native lipid A.^14^ Accordingly, a shift of *m/z* +123 is observed. Using the optimized MALDIxin test, we could (i) easily predict colistin resistance in *K. pneumoniae* by checking whether any modified (l-Ara4N or pEtN) lipid A is present in the bacterial membrane, but also (ii) discriminate between chromosome- and *mcr*-encoded resistance by looking at the percentage of l-Ara4N or pEtN modification in the modified lipid A. As expected, 100% l-Ara4N modification was observed in the case of chromosome-encoded resistance while close to 100% modified lipid A was related to pEtN addition in the case of MCR expression. Although only one isolate was available, detection of concomitant mechanisms (MCR production + disruption of chromosome-encoded MgrB) repeatedly resulted in a mixture of pEtN- and l-Ara4N-modified lipid A (about 50%/50%). In addition, despite the fact that the MALDIxin test was able to accurately detect colistin-resistant isolates, there was no strong correlation between the modification level of lipid A and the resistance level of colistin in terms of MIC.

In the context of MCR-related colistin resistance, molecular biology is widely used for the detection of MCR-producing isolates.^15^ However, the increasing number of *mcr* variants (*mcr-1* to *mcr-9*) that do not share a strong nucleotide identity will inexorably lead to false-negative results. By targeting the pEtN modification of lipid A, which corresponds to the result of all MCR variants, the MALDIxin test might be an accurate screening test for the identification of a new MCR variant.

To the best of our knowledge, this is the first MALDI-TOF MS-based method that allows the rapid detection of colistin resistance and at the same time discrimination between chromosome-encoded and MCR-related polymyxin resistance in *K. pneumoniae* without necessitating any complex lipid extraction steps. Indeed, Liang *et al*.^16^ recently described another MALDI-TOF MS-based method that has the ability to differentiate colistin-susceptible from colistin-resistant *K. pneumoniae*, but that requires fastidious sample preparation of membrane lipids with incubation in a special buffer, incubation in cooled ice, washes in ethanol and final extraction in chloroform/methanol/water (12:6:1, v/v/v).

We should acknowledge that MALDI-TOF MS analysis was performed on a 4800 Proteomics Analyzer, which is not commonly available in clinical microbiology laboratories. In addition, samples were analysed by operating in the negative ion mode of the mass spectrometer, which is not currently and widely usable on routine mass spectrometers. Accordingly, a few optimization steps are still needed to implement this test in routine use.

## Funding

This work was partially funded by the University Paris-Sud, France. Laurent Dortet is a member of the Laboratory of Excellence in Research on Medication and Innovative Therapeutics (LERMIT) supported by a grant from the French National Research Agency (ANR-10-LABX-33). Gerald Larrouy-Maumus was supported by the Department of Life Sciences in the Faculty of Natural Sciences Imperial College London, UK, and MRC-Confidence in Concept grant number 105603/Z/14/Z.

## Transparency declarations

Laurent Dortet, Alain Filloux and Gerald Larrouy-Maumus are co-inventors of the MALDIxin test for which a patent has been filed by Imperial Innovations. All other authors: none to declare.

### Author contributions

Laurent Dortet and Gerald Larrouy-Maumus had full access to all of the data in the study and take responsibility for the integrity of the data and the accuracy of the data analysis. Study concept and design: Laurent Dortet and Gerald Larrouy-Maumus. Acquisition, analysis or interpretation of data: all authors. Drafting of the manuscript: Laurent Dortet, Gerald Larrouy-Maumus and Alain Filloux. Critical revision of the manuscript for important intellectual content: all authors.

## Supplementary Material

dkz405_Supplementary_Data
