# Supplementary figures and images for: Optimization of the MALDIxin test for the rapid identification of colistin resistance in Klebsiella pneumoniae using MALDI-TOF MS
Source: J Antimicrob Chemother. 2019 Oct 3;75(1):110–6. doi: 10.1093/jac/dkz405 (PMC6910190; doi:10.1093/jac/dkz405)

**Supplementary data**


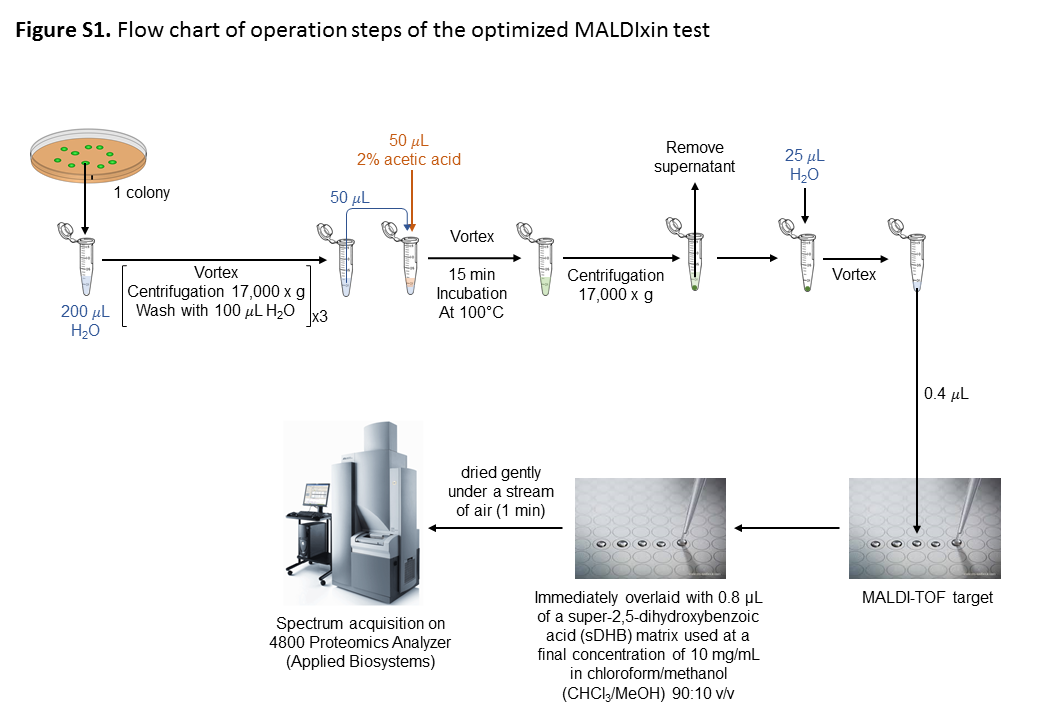

Supplement: dkz405_Supplementary_Data [file dkz405_supplementary_data.docx]
